# Combining Microfluidics, Optogenetics and Calcium Imaging to Study Neuronal Communication *In Vitro*

**DOI:** 10.1371/journal.pone.0120680

**Published:** 2015-04-22

**Authors:** Renaud Renault, Nirit Sukenik, Stéphanie Descroix, Laurent Malaquin, Jean-Louis Viovy, Jean-Michel Peyrin, Samuel Bottani, Pascal Monceau, Elisha Moses, Maéva Vignes

**Affiliations:** 1 MSC (Université Paris-Diderot, CNRS-UMR 7057), 5 Rue Thomas Mann, 75013 Paris, France; 2 Physicochimie Curie (Institut Curie, CNRS-UMR 168, UPMC), Institut Curie, Centre de Recherche, 26 rue d’Ulm, 75248 Paris Cedex 05, France; 3 Department of Complex Systems, Weizmann Institute, Rehovot, Israel; 4 Biological Adaptation and Ageing (CNRS, UMR 8256), F-75005, Paris, France; 5 Sorbonne Universités, UPMC Univ Paris 06, UMR 8256, B2A, Institut de Biologie Paris Seine, F-75005, Paris, France; University of Genova, ITALY

## Abstract

In this paper we report the combination of microfluidics, optogenetics and calcium imaging as a cheap and convenient platform to study synaptic communication between neuronal populations *in vitro*. We first show that Calcium Orange indicator is compatible *in vitro* with a commonly used Channelrhodopsine-2 (ChR2) variant, as standard calcium imaging conditions did not alter significantly the activity of transduced cultures of rodent primary neurons. A fast, robust and scalable process for micro-chip fabrication was developed in parallel to build micro-compartmented cultures. Coupling optical fibers to each micro-compartment allowed for the independent control of ChR2 activation in the different populations without crosstalk. By analyzing the post-stimuli activity across the different populations, we finally show how this platform can be used to evaluate quantitatively the effective connectivity between connected neuronal populations.

## Introduction

Behind the apparent simplicity of dissociated neuronal cultures lies an incredibly rich catalogue of behaviors present *in vivo*, including growth and differentiation [1, 2], plasticity [3, 4], electrical activity [5–10] and information processing [11–13]. The culture of primary neurons, extracted from healthy or diseased animals, has thus rapidly become an invaluable tool to understand the nervous system, from the molecular actors of neuronal functions, to the behaviors produced by embodied cultures. Interfacing such cultures is however a necessary step to understand many of these processes. For this purpose, Microelectrodes Arrays (MEAs) have remained the favorite alternative.

MEAs have excellent temporal resolution, extended coverage, and their non invasiveness makes them particularly suited for long-term experiments. While originally low, their spatial resolution has significantly improved through recent advances [14]. On the down-side, combining MEAs with micro-compartments, although possible [15–18], is unpractical as it requires alignment steps with the electrodes for each sample, while the costs and technical efforts to set up a MEA platform are substantial and consequently not adapted to non-specialized laboratories. These limitations can however be circumvented using optical methods to excite neurons and record neuronal activity as an alternative to MEAs.

On the one hand, the field of optogenetics has developed alternative methods to control neuronal activity by transducing genes coding for light-gated ion channels into neurons [19–22]. Depending on their nature and the associated ionic flux, photo-activating the channels depolarizes or hyperpolarizes the membrane, and ultimately induces or inhibits action potentials in the modified neurons. Using structured light [23, 24] or LED arrays focused on the specimen [25] allows for spatial resolution superior to those achievable with MEAs, admittedly at the expense of sophisticated optical systems and immobilized samples.

On the other hand, since action potentials in neurons go along with a calcium influx, it is possible to indirectly measure the activity of neurons by calcium imaging using standard fluorescence microscopy [26–30]. Combining these two aspects is appealing, since it opens the route to a contact-less fully optical control and monitoring of neurons activity. However, using light to concurrently stimulate and monitor neuronal activity requires the spectral properties of the stimulating channel and the calcium imaging read-out to be compatible in order to avoid constant background stimulation during imaging. For instance, Channelrhodopsin-2 (ChR2) variants excited with blue light and Fluo-4 are the most popular candidates for optogenetic stimulation and calcium imaging, respectively [31, 32], but the two are not compatible according to the aforementioned criterion. Several ChR2 variants with red-shifted excitation peaks [20] have been developed, but they remain substantially activated by blue light due to their large absorption peaks. Using UV-shifted calcium indicators (typically Fura-2) in combination with ChR2 [33] is not an optimal solution either, due to the harmful consequences of prolonged UV exposure on the cells. Finally, some groups have demonstrated *in vivo* the possibility to use a standard ChR2 variant in combination with a red-shifted calcium indicator [29, 30].

Besides the stimulation and recording aspects, the experimental toolbox for neuronal cultures has been recently enriched by new nano- and micro-technologies allowing the reconstruction of neuronal pathways under controlled geometry [34–37] and with oriented synaptic connections [38–44], opening new routes for studying cognitive processes *in vitro* [13, 40, 45, 46]. Geometrical constraints, whether imposed on single neurons or on whole networks, can furthermore facilitate observations in contexts where spatial resolution is a limiting factor.

In this paper, we demonstrate the possibility of combining optogenetics, calcium imaging and microfluidics to study neuronal connectivity. First we report that the Calcium Orange indicator, which spectrum is red-shifted compared to Fluo-4, is a suitable tool to record the activity of neuronal cultures transduced with ChR2 for extended periods of time. Secondly we evaluate the possibility to combine optical fibers and micro-compartmented cultures to increase the spatial resolution and the specificity of stimulation. Finally, we demonstrate the ability of our platform to measure functional connectivity between small neuronal populations connected through different types of micro-channels.

## Materials and Methods

### Micro-fabrication

**Fig 1:**
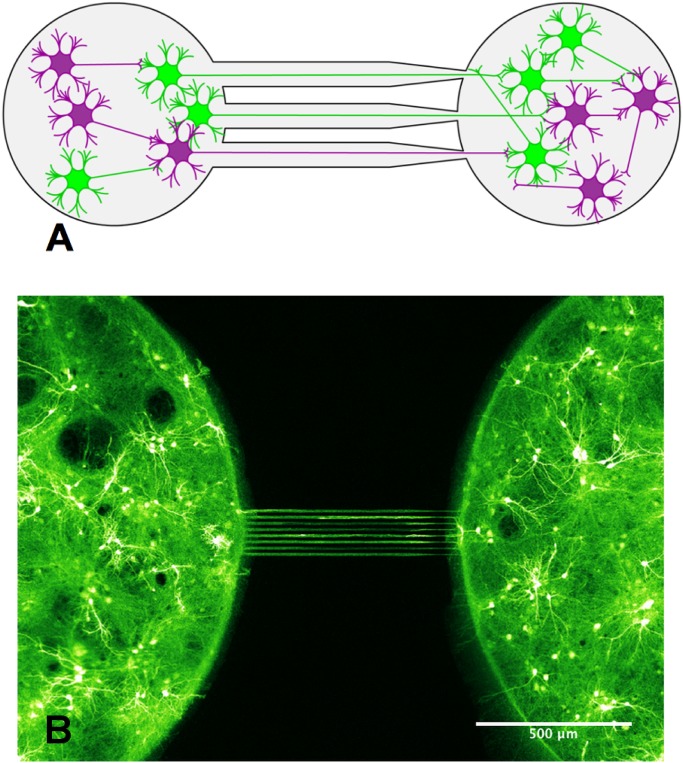
Design of a simple neuronal device. A- Scheme showing the neuronal device featuring two micro-wells seeded with hippocampal neurons (DIV 15) and connected by an array of axon diodes. The neurons colored in green correspond to neurons that are transduced by ChR2. Neurons colored in purple are not transduced. B- Image of a neuronal device obtained by confocal fluorescence microscopy showing neurons expressing ChR2-YFP (false color).

We used PDMS-on-glass chips consisting in micro-wells (2 mm in diameter) in which neurons were seeded, with bundles of micro-channels (Fig 1) connecting them. The low height (3 μm) of micro-channels was set so as to prevent cell somas from invading them, while their length (500 μm) allowed neurites to differentiate into axons and connect the different compartments together. While straight micro-channels insured symmetrical connectivity, funnel-shaped channels (15 to 2 μm) favoured axonal growth in the narrowing direction [42]. This asymmetric design, called “axon-diode”, uses a simple geometrical selection: growing axons have a higher probability to enter a wide entrance than a narrow one in the chambers wall. Once in the channel however, they essentially continue to grow with the same speed and efficiency, irrespectively of the small slanting associated with the asymmetry of the channel.

Master molds were created by spin coating a silicon wafer with a first 3 μm thick layer of SU-8 2005 (Microchem) and were then exposed with a first photomask so as to create the axon-selective micro-channels. A second layer of SU-8 3035 was spun at low speed on top of the axon channels and exposed with a second photomask in order to create pillars of 2 mm in diameter and 1 mm tall. Although we don’t present them here, we successfully created with this method smaller pillars of 1 and 0.5 mm in diameter. These pillars constitute the templates for the micro-wells. Master molds were replicated in PDMS (Sylgard 184, Dow Corning, 10:1 volume ratio) using two cycles of silanization, casting and curing. The parts to be silanized were first treated with plasma to activate their surfaces (30 s air plasma 600 mTorr at 18W, Harrick Plasma), placed in a petri dish with a drop of silane (1H,1H,2H,2H-Perfluoro-octyl-trichlorosilane, ABCR) deposited on the cover, and incubated 15 min at ambient pressure and temperature. PDMS molds are more resistant to casting-peeling cycles and can be produced easily from the intermediate PDMS counter molds, reducing clean room time and costs tremendously.

The PDMS molds were silanized before the first use and spin coated with fresh PDMS so that the top of pillars from the mold emerge. This step is critical as overflowing the pillars will yield closed micro-wells in the final product. In the event that the micro-wells are closed, they might be opened by simply tearing the PDMS membrane off the wells with clean tweezers. The PDMS parts featuring the wells and micro-channels were assembled on clean glass coverslips (18 mm diameter No.0) using plasma bounding (45 s air plasma 600 mTorr at 18 W, Harrick Plasma). After plasma activation of both coverslip and PDMS, a thin film of Poly-L-Lysine (PLL, Sigma) was created on the coverslip by adding 10 μL of 0.01% PLL, and the PDMS was deposited on the coverslip immediately. Mild pressure insured good contact between PDMS and glass while rejecting the excess of PLL in the wells and micro-channels. Curing 15 min in an oven at 70°C allows the PDMS to attach covalently to the coverslip and the PLL to dry. The microchips were rinsed thoroughly 3 times with double distilled water (DDW) to remove excess PLL, detrimental to neurons. During the rinsing step, it is important to prevent resuspended PLL from adsorbing on the top surface of the PDMS, since this would create unwanted routes for axonal growth. The rinsed chips were finally incubated with plating medium in 12 well plates (1 mL per well) for at least one hour before seeding.

### Ethics statement

Animal care and experiments were conducted in accordance with standard ethical European Committee Guidelines on the Care and Use of Laboratory Animals and the guidelines published by the Institutional Animal Care and Use Committee of the Weizmann Institute of Science (IACUC of the Weizmann Institute of Science). Experiments were approved by the IACUC of the Weizmann Institute of Science (WIS application 00420113-2) and were performed in an authorized establishment under the supervision of authorized investigators. Pregnant rats were ordered from Harlan (Rehovot, Israel). Animals were sacrificed the day of arrival using an intraperitoneal injection of pentobarbital followed by cervical dislocation.

### Neuronal cell culture

Primary neurons were obtained from rat embryos at stages E19. Dissection and papain dissociation of hippocampi was done according to established protocols[47]. Briefly, brains from embryos were micro-dissected on ice in L-15 medium without phenol red (Life technologies) supplemented with 0.6% glucose and 0.2% gentamycin. Hippocampi were digested in papain solution (papain 100 units, DNAseI 1000 units, L-Cystein 2 mg, NaOH 1M 15 μL, EDTA 50 mM 100 μL, CaCl_2_ 100 mM 10 μL, dissection solution 10 mL) at 37°C for 20 minutes. After digestion, the supernatant was carefully removed and replaced with 10 mL of plating medium (MEM without glutamine supplemented with 0.6% glucose, 1% GlutaMAX, 5% Horse Serum, 5% Fetal Calf Serum and 0.1% B27) supplemented with 25 mg of trypsin inhibitor and 25 mg of Bovine Serum Albumine for 5 min to inactivate the papain. Supernatant was removed, replaced with plating medium and tissues were triturated with fired polished pasteur pipettes. The medium covering the microchips was aspirated and the dissociated neurons were seeded in compartment at a density around 5000 neurons/mm^2^. The seeded chips were incubated half an hour in a humidified, 37°C and 5% CO_2_ incubator to allow the attachment of neurons to the PLL substrate. Each chip was finally covered by 1 ml of serum-free medium (Neurobasal, B27 4%, GlutaMAX 1% and FCS 1%) and the cultures were placed back into the incubator.

Glial proliferation was stopped 4 days after plating by adding in the medium 20 μg/mL 5-fluoro-2’-deoxyuridine and 50 μg/mL uridin (Sigma, Israel). All media contained 50 μg/mL gentamicin so as to minimise contaminations.

### Viral infection

Neurons were transduced with an Adeno-Associated Virus (mixed serotype AAV1-2) containing a pAAV-CaMKIIa-ChR2(E123T/T159C)-P2AE-YFP construct kindly given by the group of Pr. Ofer Yizhar. Due to the low cytotoxicity of AAV, we used a saturating amount of viral particles (extracted from culture supernatant) in accordance to the amounts used in Pr. O. Yizhar lab, yielding a fraction of ChR2-YFP positive neurons of around 70%. Cultures were infected by adding the virus directly to the wells a few hours after seeding. Experiments were performed at least 5 days after infection.

### Calcium imaging

The 50 μg of Calcium Orange indicator provided in each tube of the commercial AM Ester (Molecular Probe product) were resuspended into 10 μL of anhydrous DMSO to yield ∼ 4 mM stock solutions. Calcium Orange stock solutions were diluted into filtered Recording Buffer (NaCl 129 mM, KCl 4 mM, MgCl_2_ 1 mM, CaCl_2_ 2 mM, Glucose 10 mM, HEPES 10 mM) to obtain a loading solution with a final concentration in calcium indicator of 5–10 μM. The culture medium was replaced with loading solution and the cultures were incubated at room temperature for 40 minutes in the dark. The loading solution was then removed and replaced by fresh recording buffer, and the cultures were allowed to recover at 37°C for 30 min in the dark before imaging. This step also facilitates hydrolysis of the internalized AM ester precursor into its active form. Images were acquired with a scanning confocal microscope (Leica SP5) and a white-light laser set to 549 nm was used to excite the Calcium Orange. The scanning line was 1 by 256 pixels, with a pixel size of 4 μm by 4 μm. The line was positioned parallel to the axon micro-channels, astride the two populations (S1 Fig). Either end of the line was overlapping its corresponding population by ∼ 35 pixels, that is, a 4 μm by 140 μm area, over which fluorescence was integrated. The laser was exciting the line in a bidirectional way at a frequency of 8 kHz. The fluorescence was integrated over 8 cycles for each data point, resulting in a final sampling rate of 1 kHz.

### Stimulation

Optical stimulations were performed with a custom apparatus consisting of blue (470 nm) Rebel LEDs with 10 mm Square CoolBases (70 lm at 700 mA, LUXEON STAR LEDs). The LEDs were mounted on an aluminum heat-sink with thermal tape. MOSFETs (IRF840) controlled with an Arduino Leonardo board were used to achieve fast modulation of the LED forward currents (from 0 to 700 mA). The LEDs were butt coupled to 0.5 mm diameter PMMA optical fibers with a homemade adaptor keeping the fibers aligned with the LEDs. Home-made spacer and adapter were used to position the other end of each fiber on top of the different neuronal populations, at a distance of approximately 1 mm from the neurons. Since the acceptance half-angle of the PMMA fibers we used is about 26°, the spot receiving the vast majority of the light was about 1.5 mm in diameter. Each 0.5 mm fiber delivered about 3 mW of light in this configuration, as measured with a laser power meter (Ophir, Israel).

## Results and Discussion

### *In vitro* compatibility of optogenetic stimulation with calcium imaging

**Fig 2:**
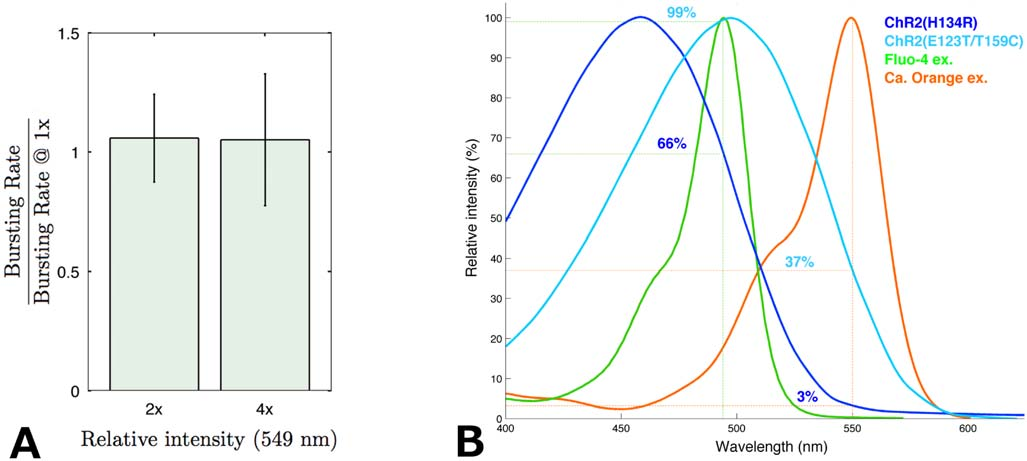
Compatibility of ChR2 and Calcium Orange. A- Quantification of background stimulation in ChR2(ET/TC) transduced cultures during calcium imaging with Calcium Orange at DIV12. The abscissa indicates the intensity of excitation light (549 nm) used during imaging relative to the recording conditions used in the rest of the experiments (1x); 2x is twice that level and 4x is 4 times that level. The different bursting rates at 2x and 4x were normalized by the bursting rates recorded at 1x for each culture so as to remove inter-culture variability for initial bursting rates. For information purpose, average absolute bursting rate at 1x was 5.9 ± 2.2 bursts per minute (n = 9 cultures). B- Absorption spectra of two ChR2 variants, including the ChR2(ET/TC) used in this study, as well as those of the commonly used Fluo-4 and its red-shifted counterpart Calcium Orange indicator. The figures of merit of each combination are indicated in % of maximal absorption.

To be able to combine optogenetic stimulations of neurons with calcium imaging, we needed to select a red-shifted calcium dye and check if the excitation of this dye did not trigger neuronal activity in population of neurons expressing ChR2. The calcium dye we have selected, Calcium Orange indicator from Molecular Probe, presents a maximum of excitation at 549 nm (Fig 2(B)). The minimum light intensity at 549 nm (obtained with a white-light laser) required to yield a sufficient signal to noise ratio was first established on non transduced cultures as a starting point for experiments.

The effect of increasing intensities of 549 nm light on ChR2 was then investigated on cultures expressing ChR2-E123T/T159C. Recorded bursting rates were normalized by the bursting rate observed under minimal light on the same culture, so as to exclude inter culture variability in term of basal activity. The results showed that activity was largely unaffected by light at 549 nm. Increasing the intensity of excitation light by 400% (compared to the reference level) yielded no significant increase (paired t-test: p > 0.25,n = 9) in the frequency of bursts (Fig 2(A)). This shows either that despite a broad absorption peak, ChR2 is not activated, or that neurons compensate for ChR2 currents through some homeostatic mechanism. However, the fact that we could induce bursts reproducibly using pulses of light at 470nm (see below) during calcium imaging shows that most of the ChR2 channels are in a responsive, thus unactivated state under such conditions.

Based on the theoretical figures of merit, other ChR2 variants (such as H134R presented in Fig 2(B)) have been considered as they could tolerate even higher levels of light at 549 nm without background stimulation of neurons (Fig 2(B)). However the maximal light intensity used to excite calcium indicators is still limited by other factors, like bleaching or phototoxicity, so that in practice the advantage of using other variants might not be as great.

### Short stimulations ignite bursting events in transduced cultures

**Fig 3:**
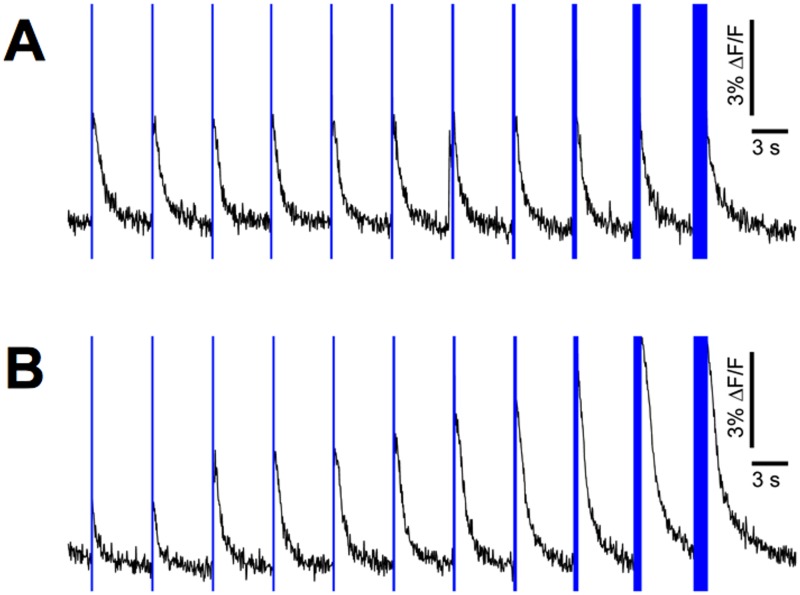
Optogenetic induction of bursting events. A- Typical responses of a transduced culture (DIV 13) to different stimulation durations (1, 2, 4, 8, 16, 32, 64, 128, 256, 512 and 1024 ms). Vertical blue bars represent stimulation times and durations. No fluorescence data was recorded during the stimulations as the photodiode, recording directly the stimulated spot, was saturated. B- Same experiment as in A after addition of 10 μM CNQX (AMPA receptor antagonist).

We characterized the response of transduced neurons to brief pulses of light (from 1 ms to 1024 ms), at 470 nm and for a dose of about 3 mW/mm^2^ (Fig 3). Stimulations were performed every 5 seconds, a rate low enough to avoid possible short term plasticity effects but also high enough to reduce the probability of occurrence of spontaneous bursts (a inter-burst interval of 5 to 10 seconds is common at 13 DIV). Note that in the case of Fig 3 where the temporal resolution was not the most critical aspect of the experiment, we binned the data by chunks of 50 ms in order to yield less noisy curves.

It is important to notice that two mechanisms are involved in the activation of neurons in these responses. The first one is the direct activation by light (via ChR2) which thus applies only to transduced neurons; the second one is an indirect activation by synaptic transmission and it applies to all neurons. To better characterize the contribution of each of these two mechanisms, we did experiments in the presence and absence of CNQX.

CNQX is an antagonist of AMPAR, a ionotropic transmembrane receptor for glutamate that mediates most excitatory transmission at synapses. In consequence, under saturating concentration of CNQX, excitatory synaptic transmission is blocked and the observed response only represents the fraction of neurons that is directly activated by light.

In the absence of CNQX we observed that each stimulation elicited a bursting event, the amplitude of which, as recorded using calcium imaging, was largely independent from the stimulus duration (Fig 3(A)). Notice that in spite of our fairly high stimulation rate, a burst does occur spontaneously just before one of the stimulations. However it appears that another burst is evoked at the actual stimulation time. It seems therefore that the stimulations are strong and override any possible natural post-burst refractory period.

After the addition of 10 μM CNQX (determined experimentally as the lowest saturating concentration), the response to the shortest stimulus was significantly reduced compared to normal condition, and increased gradually with the stimulus duration (Fig 3(B)) in contrast to the former case. This shows that under normal conditions, the amount of neurons originally activated by light is actually dependent on stimulus duration although synaptic activation eventually takes over and leads after a percolation process to a same final state.

Surprisingly, responses to longer stimulations were consistently higher in the presence of CNQX. This paradoxal finding could be explained by the simple hypothesis that inhibitory neurons do not express ChR2. This hypothesis is supported by the observed mutually exclusive marking of GAD2-positive (inhibitory) and ChR2 positive neurons (S2 Fig), in line with former work [48] reporting that the CaMKII promoter (used in our experiment to express ChR2) is significantly less active in inhibitory neurons than in excitatory neurons. During stimulation in the presence of CNQX, the inhibitory neurons would remain silent because their sole excitation pathway is blocked. While the inhibitory neurons (10–20% of neurons in culture) would not contribute to the collective response anymore, the rest of the culture, uninhibited, would be able to respond more strongly upon ChR2 activation.

The possibility that the first neurons to respond might be only excitatory would change the modality of recruitment of neurons in the burst. It therefore raises the more general question of whether individual neurons experience evoked bursts differently from endogenous ones based on the timing of activation of their pre and post-synaptic partners. Although our data does not allow us to conclude, other groups have developed complementary methods to monitor fast propagation of neuronal activity in small neuronal populations[45, 46]. Such methods could help us determine whether evoked activity is fundamentally different from spontaneous one, and understand the implications of stimulations regarding possible plasticity and adaptation effects in cultures.

### Locally induced bursts propagate through axon channels

**Fig 4:**
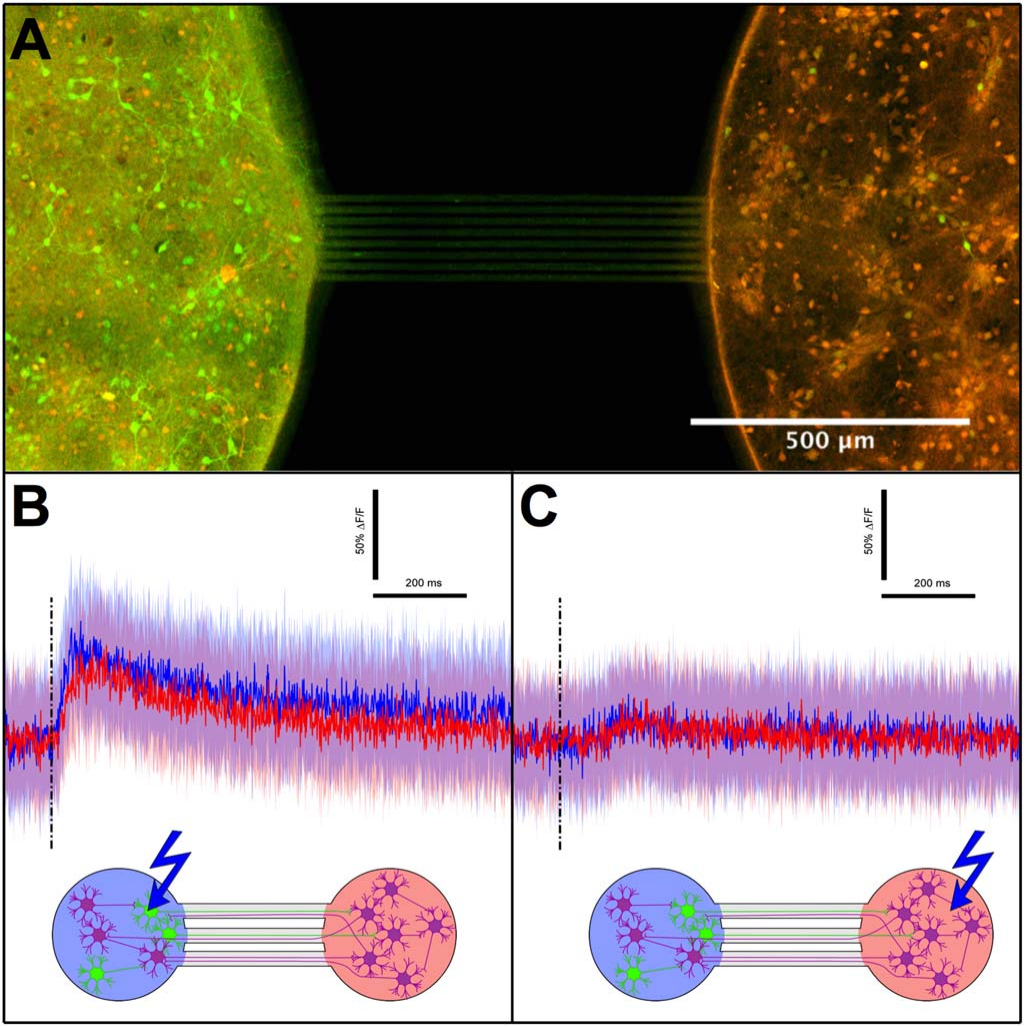
Local stimulation and burst propagation (DIV15). A- Picture of a symmetrical two-compartments device with only one side transduced (left). Green and red channels are for ChR2-YFP and Calcium Orange indicator, respectively. This dual marking reveals that 70% of cells are expressing ChR2 inside the transduced compartment. B and C- Averaged response after stimulation of the transduced (B) and non-transduced (C) compartments. The solid colour lines correspond to the peristimulus fluorescence signal averaged over 30 stimulations (0.1 Hz in alternation) while the filled surfaces indicate associated standard deviations. Vertical dashed lines indicate stimulations. Diagrams are presented for clarity, with matching line and population colors. Green neurons are transduced neurons.

In order to study neuronal functional connectivity, we used a system in which two populations (contained in 2 mm diameter chambers) were connected by symmetrical micro channels, but only one population was expressing ChR2 (Fig 4(A)). To independently control the illumination of the two populations, we developed a system based on optical fibers in which each fiber is coupled to an LED on one end and a neuronal population on the other end, and in which each LED comes with an independent control circuit.

Stimulating the transduced side with 5 ms pulses reproducibly evoked bursts in the transduced population, as well as in the non transduced population (Fig 4(B)). As non transduced neurons do not directly respond to light, we concluded that functional synapses between the two populations must have formed, allowing activity to propagate from the transduced side to the non transduced side. This transmission of activity is in accordance with results from Peyrin et al. who already demonstrated that neuronal activity can propagate through axons grown in micro-channels [42].

In contrast, neither side produced a bursting event when the non-transduced population was stimulated. In some cases like presented on Fig 4(C) we observed a minimal response in both populations probably due to the activation of a subnetwork too small to ignite actual bursting events. Since in all cases, light diffusing from the other side was not able to ignite a burst in the transduced population, we concluded that there is no light-induced crosstalk between the two chambers in our system.

### Axon diodes produce asymmetrical functional connectivity

When both populations are transduced, the absence of crosstalk previously demonstrated guarantees that post-stimulus activity evoked reproducibly in the non stimulated population is due to synaptic transmission only. We thus applied our system to a pair of transduced populations in order to evaluate transmission independently in each direction, by initiating a burst on either side and measuring the response in the opposite side over 30 repetitions, so as to average out any non specific response (like spontaneous activity occurring by chance in the peri-stimulus time window). Thanks to the short data collection window allowed by line-scanning (see methods), we could record the response to stimulations in each chamber with a sub millisecond resolution. We could thus evaluate the delay of transmission from one population to the other, by maximizing their normalized cross correlation coefficient. Chips with straight channels produced a strong and symmetrical connectivity, where the delay of transmission was systematically smaller than 5 ms (Fig 4(B)) suggesting a high reciprocal connectivity between the two populations.

**Fig 5:**
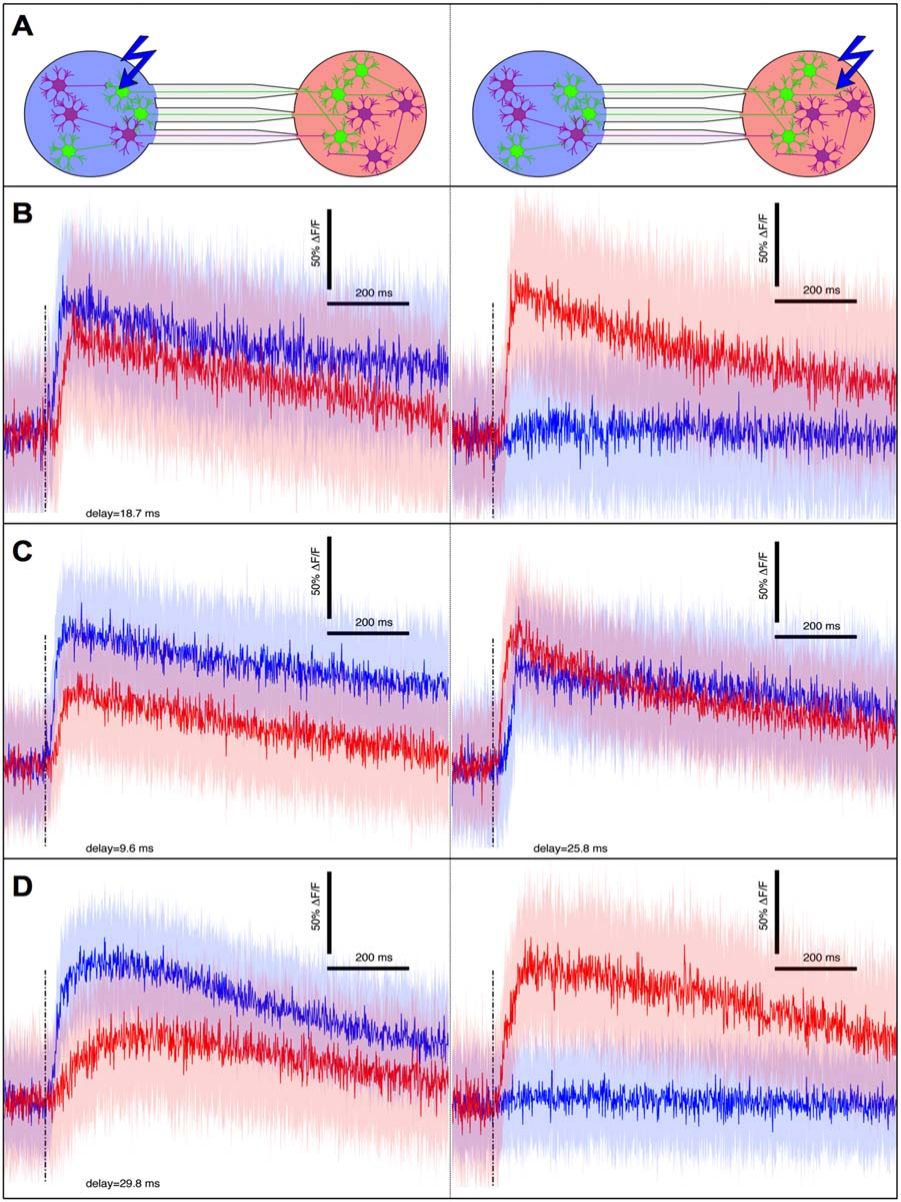
Burst transmission in asymmetrical networks (DIV15). A—Explanatory diagrams for graphs presented on the left and right column respectively, with the same color code as in Fig 4. B—Typical example of a device showing unidirectional transmission. C—Results from a device where bursts also propagate in the reverse direction, albeit with a greater delay. Addition of 5 μM CNQX to the latter device yields the typical unidirectional transmission (D). Delays obtained by normalized cross correlation.

In a second approach we used narrowing micro-channels called ”axon-diodes”, already known to orient axonal growth and synaptic transmission [42] (see methods for details), and we observed a strong functional polarity of the neuronal connectivity. In one third of all cases (n = 15 pairs), activity exclusively propagated in the forward (or narrowing) direction of the axon channels (Fig 5(B)). In the other cases, reverse transmission also occurred, albeit with a significantly greater delay (paired t-test: p < 0.005,n = 10) with a relative increase comprised between 23% and 211% (Fig 5(C)). We interpreted this increased delay as the time necessary to integrate enough signals, from a reduced number of axons, to ignite a burst. The fact that reverse transmission could occur is not totally surprising for two reasons. The relative fraction of axons growing in the reverse direction is not negligible; it has been previously estimated to be around 3% [42]. Secondly the minimal number of activated neurons to ignite a burst is apparently small according to the results presented in Fig 3; only a few axons would be necessary to relay a burst.

Interestingly, reducing the overall excitatory neurotransmission by adding onto these cultures an under-saturating concentration of 5 μM CNQX restored an absolute unidirectional transmission (Fig 5(D)). Our interpretation is that CNQX increases the threshold of burst initiation to the point that the reverse connectivity becomes insufficient while the forward connectivity, originally stronger, can still carry enough signals to trigger a burst in the output population. These threshold effects and their link to synaptic strength can be explained by the quorum percolation model[49]. It is clear that the presence or lack thereof of burst transmission on the one hand, and the delay in burst transmission on the other hand, are two sides of the same coin, namely, functional connectivity.

These experiments show that the functional connectivity yielded by axon diodes is highly polarized and confirm the previous results of Peyrin et al. [42]. The results also suggest how delicate it is to maintain an absolute unidirectional transmission when the residual reverse connectivity is amplified by percolation effects. Luckily in case of bidirectional transmission, delay measurements still provide information about differential connectivity. We thus believe that this functional approach offers a new and easy way to evaluate quantitatively and with great sensitivity the synaptic connectivity between neuronal populations, complementing direct synaptic imaging and electrophysiology.

## Conclusion

In this paper, we have described an experimental setup, combining optogenetics, calcium imaging and microfluidics, as a tool to measure functional connectivity between different populations of neurons in reconstituted networks.

The optical recording and stimulation method used in this setup has by itself significant advantages over micro-electrode arrays. Besides alleviating the need for micro-electrodes fabrication technologies and tedious alignement of electrodes with micro-patterned substrates, the method allows for a fully non-contact system where all the sophistication of the measurement is retained in the non-disposable optical components of the instrument, and all the elements in contact with neurons are disposable, reducing the costs and facilitating sample preparation. In systems like the presented micro-chips, where a high spatial resolution is not necessary, a temporal resolution comparable to that of MEAs could be achieved at a far lesser cost, using photodiodes instead of a scanner or an ultra fast camera.

The prospect of measuring effective synaptic strength without heavy and expensive electrophysiology techniques is very promising for large scale studies on synaptic plasticity, memory and their importance in neurodegenerative diseases.

With ongoing developments aiming at increasing the throughput of the experiments, by recording and stimulating multiple chips at once, a large number of parameters such as neuronal type, culture conditions, network topology, or stimulation patterns could be systematically examined to get deeper insights into fundamental aspects of neuronal networks behavior.

## Supporting Information

S1 Fig Calcium imaging acquisition process. A. The scanning line of the confocal microscope was positioned following the dashed line so that it covered both populations in roughly equal proportions. B. Resulting imaging data are x-t pictures, from which we extracted the fluorescence traces presented in Fig 4 and Fig 5. The dashed lines delimit the two regions over which fluorescence was integrated. A stimulation initiated on the right side is visible in this recording, and appears as a blue stripe.
(PNG)

S2 Fig GAD2 inhibitory neurons do not express ChR2. The image shows a primary culture of neurons extracted from GAD2-Cre × ROSA:loxp-stop-loxp:tdTomato embryos (JAX 010802 and 007909 respectively). Only the GAD2-positive inhibitory neurons expressing the Cre recombinase can excise the floxed stop codon and express tdTomato (in red). It appears clearly that ChR2-YFP (green), controlled by the CamKIIa promoter, is not expressed in GAD2-positive inhibitory neurons.
(PNG)
