# Supplementary figures and images for: Combining Microfluidics, Optogenetics and Calcium Imaging to Study Neuronal Communication In Vitro
Source: PLoS One. 2015 Apr 22;10(4):e0120680. doi: 10.1371/journal.pone.0120680 (PMC4406441; doi:10.1371/journal.pone.0120680)

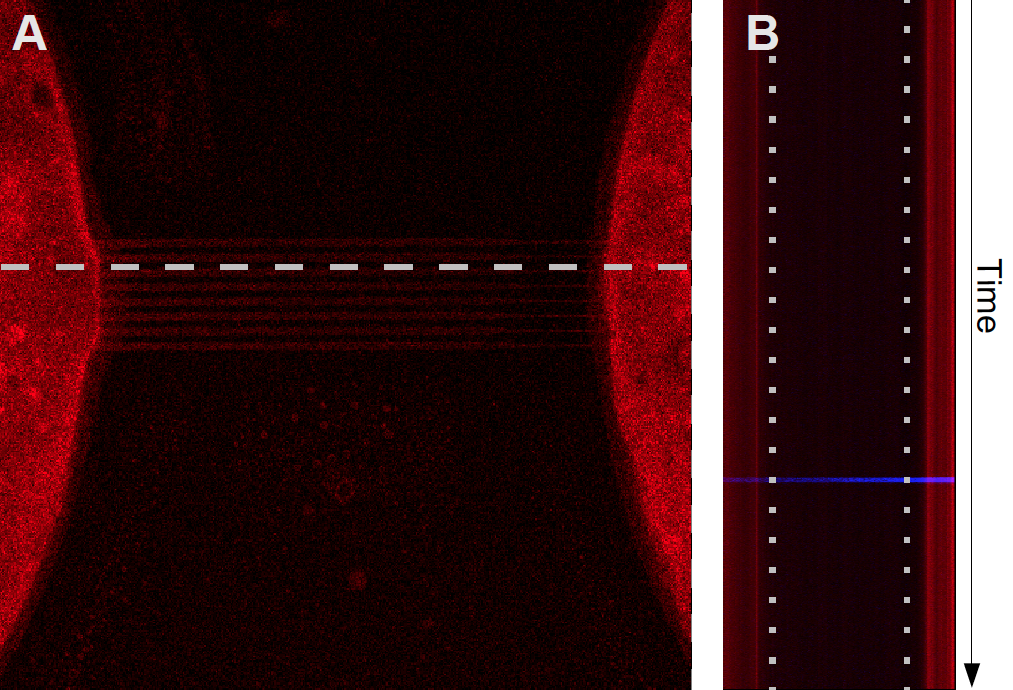

Supplement: S1 Fig — A. The scanning line of the confocal microscope was positioned following the dashed line so that it covered both populations in roughly equal proportions. B. Resulting imaging data are x-t pictures, from which we extracted the fluorescence traces presented in Fig 4 and Fig 5. The dashed lines delimit the two regions over which fluorescence was integrated. A stimulation initiated on the right side is visible in this recording, and appears as a blue stripe. (PNG) [file pone.0120680.s001.png]

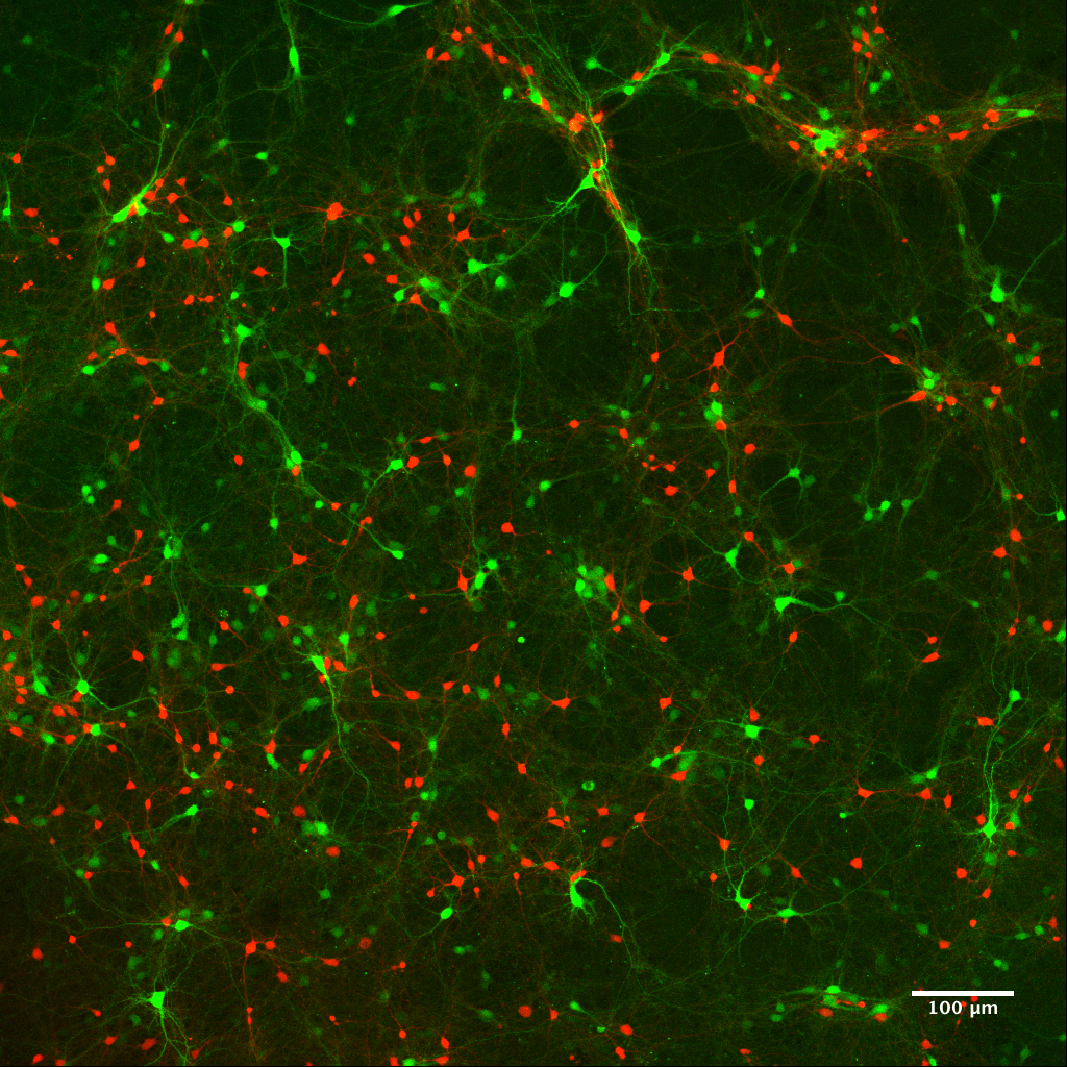

Supplement: S2 Fig — The image shows a primary culture of neurons extracted from GAD2-Cre × ROSA:loxp-stop-loxp:tdTomato embryos (JAX 010802 and 007909 respectively). Only the GAD2-positive inhibitory neurons expressing the Cre recombinase can excise the floxed stop codon and express tdTomato (in red). It appears clearly that ChR2-YFP (green), controlled by the CamKIIa promoter, is not expressed in GAD2-positive inhibitory neurons. (PNG) [file pone.0120680.s002.png]
